# Black Yeast Genomes Assembled from Plastic Fabric Metagenomes Reveal an Abundance of Hydrocarbon Degradation Genes

**DOI:** 10.1128/MRA.01459-20

**Published:** 2021-04-08

**Authors:** Osman Radwan, Oscar N. Ruiz

**Affiliations:** a Environmental Microbiology Group, University of Dayton Research Institute, Dayton, Ohio, USA; b Fuels and Energy Branch, Aerospace Systems Directorate, Air Force Research Laboratory, Wright-Patterson Air Force Base, Ohio, USA; Vanderbilt University

## Abstract

We report the assembly and annotation of 10 different black yeast genomes from microbiome metagenomic data derived from biofouled plastic fabrics. The draft genomes are estimated to be 9 to 33.2 Mb, with 357 to 5,108 contigs and G+C contents of 43.9% to 57.4%, and they harbor multiple genes for hydrocarbon adaptation and degradation.

## Abstract (precis)

We report the assembly and annotation of 10 different black yeast genomes from microbiome metagenomic data derived from biofouled plastic fabrics. The draft genomes are estimated to be 9 to 33.2 Mb, with 96 to 1,257 contigs and G+C contents of 43.9% to 57.4%, and they harbor multiple genes for hydrocarbon adaptation and degradation.

## ANNOUNCEMENT

Black yeast is a diverse group of fungi, belonging to the *Chaetothyriomycetidae* and *Dothideomycetidae* subclasses of ascomycetes, with the ability to degrade hydrocarbons and plasticizers (1–3). In this study, the metagenome-assembled genomes (MAGs) of 10 black yeast species were assembled and annotated from a shotgun metagenomic library of microbiomes associated with biofouled plastic fabric materials (4).

As described by Radwan et al., total genomic DNA was extracted from plasticized fabrics exposed to the Panama jungle for 14 months, and the DNA libraries were sequenced using a shotgun metagenomic approach (4). To maximize DNA extraction efficiency, fabrics were cut into 0.5-cm^2^ pieces, and DNA was extracted using the DNeasy UltraClean kit (catalogue number 12224-250; Qiagen) (4). DNA libraries were constructed using the PrepX DNA library kit and the Apollo 324 next-generation sequencing (NGS) automatic library preparation system (WaferGen, Fremont, CA). The TruSeq paired-end DNA libraries were sequenced using an Illumina HiSeq 2000 system, generating 161,537,275,209 bp of raw reads of 100-bp length (4). Trimmomatic v0.36 with the RepBase-20170 library (5) was used for quality control, removing raw reads shorter than 50 bp and those with an average quality score below 15. Multiple bioinformatics programs (6) were used with default parameters except where otherwise noted. After sorting of paired-end reads for compatibility and normalization using BBtools (https://jgi.doe.gov/data-and-tools/bbtools), reads were assembled by the MEGAHIT assembler (7) with options of minimum contig length of 200 bp and meta-sensitive. Fasta contigs produced by MEGAHIT with coverage and abundance files for each fabric sample were used by MaxBin v2.7.7 (8) to bin individual genomes with options of minimum contig length of 2000 and depth of 2.

DNA sequences of 10 different black yeast genomes were selected for assembly improvement and sequence gap filling using SSPACE v3.0 (9) and GapFiller v1.10 (10), respectively (Table 1). After masking of the repetitive sequences with RepeatMasker (11), the genes of each genome were predicted by AUGUSTUS v2.5.5 (12) with an option set for Yarrowia lipolytica. CEGMA (13) was used to calculate genome completeness, which ranged from 13.64% for Hortaea werneckii to 94.00% for Exophiala oligosperma. According to the MAG completeness criteria (14), two MAGs were high quality, two were medium quality, and six were low-quality drafts reflecting partial genomes. ABySS v2.0.2 (15) calculated genome sizes ranging from 9 to 33.2 Mb, with *L*_50_ values of 22 to 1,258 contigs and G+C contents of 43.9% to 57.4% (Table 1). HMMER v3.3 (16) with E values of 1*e*^−3^ was used to search the Pfam database for functional annotation of proteins involved in hydrocarbon degradation (i.e., cytochrome P450 and aromatic ring-opening dioxygenases), hydrolases (esterases and lipases), and efflux pumps potentially associated with hydrocarbon resistance (Table 1). The number of protein-coding genes identified for each pathway depended on the assembled genome completeness and species. For example, Exophiala oligosperma, with a genome completeness of 94.00%, contained the highest number of proteins belonging to different pathways, while Hortaea werneckii, with only 13.64% completeness, contained the lowest number of proteins. The results of this study support the ability of black yeasts such as Exophiala oligosperma and Cyphellophora europaea to degrade hydrocarbons (17, 18) and plasticizers (1, 2).

**TABLE 1:** Accession numbers, general statistics of black yeast MAGs, and numbers of genes involved in hydrocarbon degradation, polymer hydrolysis, and efflux pumps for each genome^*a*^

Code^*b*^	Genome	GenBank accession no.	Size (bp)	No. of contigs	*L*_50_ (contigs)	*N*_50_ (bp)	G+C content (%)	Completeness (%)^*c*^	No. of degradation genes	No. of hydrolase genes	No. of MFS^*d*^	No. of ABC^*e*^
A04	Exophiala oligosperma	JADCRK000000000	33,192,209	357	22	451,697	51.6	94.00	137	6	143	85
B02	Rhinocladiella mackenziei	JADCRN000000000	26,458,337	925	101	73,418	43.9	93.95	19	0	50	16
B03	Baudoinia panamericana	JADCRE000000000	27,253,305	5,108	1,198	6,330	49.7	68.55	112	4	125	65
B04	Cyphellophora europaea	JADCRG000000000	18,212,429	4,098	1,132	4,855	54.4	55.65	77	3	216	63
B06	*Exophiala* sp.	JADCRL000000000	20,651,700	4,626	1,258	4,953	45.8	47.58	26	0	47	20
C02	Cyphellophora europaea	JADCRH000000000	18,567,089	4,594	1,232	4,116	56.9	42.74	74	3	178	31
A11	Baudoinia panamericana	JADCRF000000000	13,998,733	3,358	935	4,505	49.9	39.52	28	3	53	13
F22	Cyphellophora europaea	JADCRI000000000	11,716,283	3,003	806	4,045	55.3	24.19	23	3	131	67
B07	Cyphellophora europaea	JADCRJ000000000	9,001,416	2,896	1,036	3,049	57.4	18.95	80	4	166	14
A17	Hortaea werneckii	JADCRM000000000	9,816,071	2,164	625	5,040	52.6	13.64	1	0	1	11

a The Pfam database with E values of 1*e*^−3^ was used for functional annotation. High-quality (A04 and B02), medium-quality (B03 and B04), and low-quality (C02, A11, F22, B06, B07, and A17) draft genomes are shown. The taxonomic identity of each species was determined using Kaiju (19).

b A04, A11, and A17 were derived from physical biosample A (BioSample accession number SAMN16111753; SRA accession number SRS7586490). B02, B03, B04, B06, and B07 were derived from physical biosample B (BioSample accession number SAMN16111857; SRA accession number SRS7586491). C02 was derived from physical biosample C (BioSample accession number SAMN16111912; SRA accession number SRS7586492). F22 was derived from physical biosample F (BioSample accession number SAMN16112156; SRA accession number SRS7586495).

c Genome completeness was estimated with CEGMA software (13).

d MFS, major facilitator superfamily efflux pumps.

e ABC, ABC transporters.

### Data availability.

The raw sequence reads and MAGs were deposited in DDBJ/ENA/GenBank under BioProject accession number PRJNA656291 with BioSample accession numbers SAMN15776667 to SAMN15776676 and SRA accession numbers SRX9364069 to SRX9364074. Individual accession numbers for MAGs are provided in Table 1. HMMER results can be retrieved from Mendeley Data at https://data.mendeley.com/datasets/kh6x88k83y/1.
